# Diet quality in relation to the risk of hypertension among Iranian adults: cross-sectional analysis of Fasa PERSIAN cohort study

**DOI:** 10.1186/s12937-021-00717-1

**Published:** 2021-06-26

**Authors:** Amir Motamedi, Maryam Ekramzadeh, Ehsan Bahramali, Mojtaba Farjam, Reza Homayounfar

**Affiliations:** 1 Student Research Committee, Shiraz University of Medical Sciences, Shiraz, Iran; 2 Nutrition Research Center, Department of Clinical Nutrition, School of Nutrition and Food Sciences, Shiraz University of Medical Sciences, Shiraz, Iran; 3 Noncommunicable diseases research center, Fasa university of medical sciences, Fasa, Iran; 4 Faculty of Nutrition Sciences and Food Technology, National Nutrition and Food Technology Research Institute Shahid Beheshti University of Medical Sciences, Tehran, Iran

**Keywords:** Mediterranean diet, Diet quality index-international, Healthy eating index-2015, Dietary diversity score, Hypertension

## Abstract

**Background:**

Hypertension is a common chronic disease with various complications and is a main contributing factor to cardiovascular disease (CVD). This study aimed to assess the association of diet quality, assessed by dietary diversity score (DDS), Mediterranean dietary score (MDS), diet quality index-international (DQI-I), and healthy eating index-2015 (HEI-2015) with the risk of hypertension.

**Methods:**

This study recruited a total of 10,111 individuals (45.14% male) with mean age of 48.63 ± 9.57 years from the Fasa Cohort Study, Iran. Indices of diet quality, including MDS, HEI-2015, DQI-I, and DDS were computed by a 125-item Food Frequency Questionnaire. Participants were diagnosed as hypertensive if they had a diastolic blood pressure (DBP) ≥90 mmHg, systolic blood pressure (SBP) ≥140 mmHg,, or used antihypertensive drugs.

**Results:**

Hypertension was prevalent in 28.3% of the population (21.59% in males and 33.74% in females). In the whole population, after adjustment for potential covariates, including daily energy intake, age, gender, physical activity, smoking, family history of hypertension, body mass index, and the level of education, higher adherence to the MDS (OR: 0.86, 95%CI = 0.75–0.99) and HEI-2015 (OR: 0.79, 95%CI = 0.68–0.90) was significantly associated with decreased risk of hypertension. The protective effect of HEI-2015 against hypertension remained significant for both males (OR: 0.80, 95%CI = 0.64–0.99) and females (OR: 0.78, 95%CI = 0.66–0.94), while, for MDS, this relationship disappeared in the subgroup analysis by gender. DQI-I and DDS were not related to the odds of hypertension.

**Conclusions:**

Adhering to MDS and HEI-2015 diets could contribute to the prevention of hypertension.

**Supplementary Information:**

The online version contains supplementary material available at 10.1186/s12937-021-00717-1.

## Background

Hypertension, as a worldwide health problem, is a main contributing factor to cardiovascular disease (CVD) and premature death, affecting about 1 billion adults globally [1, 2]. In contradiction of trends reported in the USA and northern Europe [3], evidence indicates that CVD mortality is elevating in Iran; hypertension is prevalent in 24% of women and 22% of men in this population [4]. It is anticipated that the burden of hypertension on communities rises continuously along with population ageing. Evidence has demonstrated that age, genetics, ethnicity, gender, and socioeconomic status are risk factors for hypertension [5]; nevertheless, this disease is modifiable [6, 7] and well-controlled blood pressure (BP) can enhance quality of life, improve prognosis, and prevent its clinical complications [8, 9].

The changes in lifestyle, such as physical activity, weight reduction and dietary modifications are the main approaches for the prevention and management of hypertension [10]. Excessive consumption of dietary sodium, saturated fatty acids, cholesterol, and alcohol are shown to be linked to an elevated risk of hypertension, while intake of vegetables/fruits and foods rich in magnesium, calcium, potassium, and unsaturated fatty acids is reported to reduce blood pressure [5, 11]. Whereas previous dietary recommendations for the prevention of hypertension focused mainly on single micronutrients or food items, a more effective approach for this purpose is recommendations that cover the entire quality of diet using dietary pattern, considering potential interactions of food items/nutrients [12–16]. The overall quality of diet could be assessed using the a posteriori and the a priori methods, in which the a posteriori approaches derive dietary patterns exploratory according the intake of foods reported by the studied population, but, the a priori approaches evaluate the adherence of participants to a predefined healthy dietary index [17]. Dietary approach to stop hypertension (DASH) dietary pattern, featured by low intake of salt, saturated fat, red meat, and high intake of low-fat dairy products, fish, nuts, fruits, whole grains, vegetables, magnesium, potassium, calcium, and fiber, is the most effective dietary pattern to reduce BP [18]. Moreover, evidence shows that higher diet quality assessed by other a priori dietary indices, including the Mediterranean dietary score (MDS), diet quality index-international (DQI-I), healthy eating index-2015 (HEI-2015), and dietary diversity score (DDS) are protective against the risk of CVD [19–22]. Nevertheless, the relation of these indices to hypertension is not well-established.

The Fasa Cohort Study [23] provided this opportunity to evaluate diet–disease associations from an epidemiological standpoint. The present analysis was conducted to explore the relation of MDS, HEI-2015, DQI-I, and DDS to the risk of hypertension in an Iranian population.

## Methods

### Study population

This study was cross-sectional in design, which used the data of the Fasa Cohort Study as a branch of the Prospective Epidemiological Research Study in Iran (PERSIAN) cohort [24], designed to examine factors predicting chronic non-communicable diseases. Detailed protocol of the Fasa Cohort Study is reported previously [23]. It was performed during November 2014 and June 2019. In summary, among a total of 41,000 persons resident in rural regions of Sheshdeh, a district from Fasa County, 11,097 individuals aged 35 to 70 years were included. Subjects were included if they were not physically or mentally disabled, aged 35–70 years, and lived in Sheshdeh district ≥9 months each year. All subjects had a similar socioeconomic level. For this study, after excluding 985 subjects (8.88%) due to incomplete data on dietary intake and BP as well reporting unusual daily energy intake (±3 SD of mean energy intake), a total of 10,112 subjects (7254 participants without hypertension and 2858 participants with hypertension) finally participated in the study. Subjects were invited by healthcare experts working in the rural health system to obtain blood samples, BP, dietary intake and general information. All individuals participating in the study signed a written consent. The Ethics Committee of Shiraz University of medical sciences, Shiraz, Iran approved the protocol of the study (code: IR.FUMS.REC.1399.500).

### Dietary intake assessment

A validated 125-item Food Frequency Questionnaire (FFQ) [25] was applied to assess the usual dietary intake of subjects through a face to face interview by the same expert nutritionist. The FFQ used in our study was modified in accordance with the Iranian food culture, in which participants were asked to report the frequency and amount of each food item on daily, weekly, monthly, and yearly bases through the past year. All of the portion sizes or household measures for each food were converted to grams/day of consumption. Then, energy and nutrient contents of foods were calculated using the Nutritionist IV software (version 7.0).

### Healthy eating index-2015 (HEI-2015)

HEI-2015 was calculated using the technique described by Krebs-Smith et al. [26], considering the intake of 13 dietary components including whole fruits, total fruits, total protein foods, total vegetables, seafood and plant proteins, greens and beans, whole grains, dairy products, fatty acids, refined grains, sodium, added sugars and saturated fats. Consumption of the first six components was scored between 0 and 5, while other components received a score from 0 to 10 in proportion to the intake of related foods. For the first 9 components, subjects with the highest intake were given the highest score and those consuming lowest intake received the lowest score, while for sodium, saturated fats, added sugars, as well as refined grains, the participants who consumed the highest amount of the related item were given the lowest score proportionally. The score of all components was added together to calculate the total score of the HEI-2015, ranging between 0 and 100.

#### Mediterranean dietary score (MDS)

The MDS was calculated based on the method of Trichopoulou (2003) et al. [27]. Its components include legumes, fruits and nuts, vegetables, the ratio of monounsaturated fatty acids to saturated fatty acids (MUFA/SFA), cereals, dairy products, fish, alcohol, and red and processed meats. In this study, due to the low reporting of alcohol intake in Iranians as well as the absence of alcohol in the Iranian FFQ, the modified model of the MDS was applied, in which alcohol consumption was not included in the calculations. According to the median intake of the mentioned components, a score of 0 or 1 was given to each factor as follows: for vegetables, fish, cereals, legumes, a high ratio of MUFA/SFA, and fruits and nuts, those with consumption equal to or lower than the median received score of 0, and 1 otherwise. For other components, individuals whose consumption were below or equal to the median were given a value of 1, and 0 otherwise. Finally, the scores of all the components were added together to obtain the total score of the MDS. The total score ranged between 0 and 8 and a higher score correspond to greater adherence to the MDS.

### Diet diversity score (DDS)

DDS was calculated using the protocol of Kant et al. [28] based on the intake of five food groups including bread-grains, fruits, vegetables, dairy products, and meats. These groups were divided into 23 subgroups in order to represent the dietary diversity across the groups of the Food Guide Pyramid. Bread-grains group was divided into seven subgroups (biscuits, refined bread, whole bread, macaroni, refined flour, corn flakes, and rice), vegetable group divided into seven subgroups (tomato, potato, vegetable, yellow vegetables, green vegetables, other starchy vegetables, legumes), dairy group divided into three subgroups (milk, cheese, and yogurt), fruits group divided into two subgroups (fruit juices and fruits), and four subgroups were considered for meat group (eggs, poultry, red meat, and fish). Participants were considered as consumer of one food group if they consumed at least one-half serving daily as described by the Food Pyramid quantity criteria. The maximum score of 2 was assigned to each group and the total DDS ranged between of 0 and 10.

### Diet quality index-international (DQI-I)

According to the method of Kim et al. [29], the DQI-I was estimated using four features of a healthy diet comprising moderation, balance, variety, and adequacy. The total score of DQI-I is 100 and a higher score represents a higher quality of diet.

### Assessment of other variables

BP was measured by an expert nurse 15-min resting in a sitting position (sphygmomanometer, mercury, ALPK1, Japan) and was repeated after an additional 15 min. The average of two assessments was recorded as the final BP. Participant were diagnosed as hypertension if they had a diastolic blood pressure (DBP) ≥90 mmHg, systolic blood pressure (SBP) ≥140 mmHg,, or used antihypertensive drugs. Physical activity was assessed by the International Physical Activity Questionnaire (IPAQ) and reported as metabolic equivalent hour per day (MET. h/day). Weight and height were evaluated with the use of a digital scale and body mass index (BMI) was computed by dividing the weight (kg) by height squared (m2). Waist circumference (WC) and hip circumference (HC) were measured using a constant tension tape with a precision of 0.1 cm at the level of the umbilicus and the widest point over the buttocks, respectively.

### Statistical analysis

Subjects were categorized based on the quartiles of scores of dietary indices. Differences in quantitative and qualitative variables across quartile of scores of dietary indices were evaluated using the One-way analysis of variance (ANOVA) and I2 tests for quantitative and qualitative variables, respectively. Odds ratio (OR) and 95% confidence interval (CI) for the relation of dietary indices to the risk of hypertension was computed using the binary logistic regression analysis. Model 1 was controled for daily energy intake; model 2 was adjusted for dietary energy intake as well as age and gender; and model 3 included covariates adjusted for in model 2 plus physical activity (continuous), smoking (yes/no), family history of hypertension (yes/no), body mass index, and education level (years of education). All statistical tests were conducted by using of SPSS (version 18). *P* value <0.05 was considered as significant.

## Results

A total of 10,112 individuals (45.14% male), with mean age of 48.63 ± 9.57 years, participated in this study. The prevalence of hypertension among the study population was 28.26% (21.59% in males and 33.74% in females).

It was observed that in men and women with hypertension compared with those with normal blood pressure, the means of age, weight, waist to hip ratio (WHR), BMI, SBP, and DBP were significantly higher, but their education, height, and physical activity were significantly lower. Also, the frequency distribution of individuals in terms of history of diabetes, alcohol consumption, active smoking, taking supplements, and history of ischemic heart disease were significantly higher in men with hypetension compared with men with normal BP. For women with hypertension compared with those with normal blood pressure, the frequency distribution of individuals in terms of history of diabetes, alcohol consumption, active smoking, taking supplements, and history of ischemic heart disease were significantly lower in women with hypetension compared with women with normal BP. Also, in the groups with high blood pressure, compared to the groups with normal blood pressure, the mean score of DDS, MDS, DQI-I, and HEI-2015 was lower (Table 1).

**Table 1:** Distribution of baseline variables and mean score of dietary indices in men and women with and without hypertension

		Men (*n* = 4565)			Women (*n* = 5547)		
	total	With hypertension (*n* = 986)	Without hypertension (*n* = 3579)	*P*-value	With hypertension (*n* = 1872)	Without hypertension (*n* = 3675)	*P*-value
Age (year)^a^	48.63 ± 9.57	53.81 ± 9.97	47.19 ± 9.00	≤0.001	53.62 ± 9.16	46.11 ± 8.64	≤0.001
Education (year)^a^	4.66 ± 3.88	4.99 ± 4.23	6.05 ± 4.09	≤0.001	2.51 ± 2.90	4.32 ± 3.42	≤0.001
Height (cm)^a^	161.67 ± 9.65	168.16 ± 6.44	169.19 ± 7.09	≤0.001	155.17 ± 6.60	155.92 ± 7.29	≤0.001
Wight (kg)^a^	67.02 ± 13.32	74.68 ± 13.64	67.72 ± 13.37	≤0.001	67.44 ± 12.86	64.04 ± 12.45	≤0.001
WHR^a^	0.93. ±0.06	0.94 ± 0.06	0.9 ± 0.06	≤0.001	0.96 ± 0.06	0.93 ± 0.06	≤0.001
BMI(kg/m2)^a^	25.64 ± 4.85	26.36 ± 4.34	23.59 ± 4.26	≤0.001	27.94 ± 4.94	26.28 ± 4.73	≤0.001
Physical activity(MET)^a^	41.47 ± 11.34	42.81 ± 13.67	45.84 ± 14.42	≤0.001	37.72 ± 6.49	38.77 ± 6.81	≤0.001
DBP (mmHg)^a^	74.65 ± 11.99	87.66 ± 11.81	70.69 ± 8.85	≤0.001	83.93 ± 12.37	70.28 ± 8.98	≤0.001
SBP (mmHg)^a^	111.36 ± 18.50	131.16 ± 18.29	104.83 ± 12.30	≤0.001	127.98 ± 20.12	103.94 ± 12.50	≤0.001
Marital status^b^				0.06			0.001
Non-married	1118	96 (2.7)	16 (1.6)		620 (16.9)	386 (20.6)	
Married	8994	3483 (97.3)	970 (98.4)		3055 (83.1)	1486 (79.4)	
History of diabetes^b^	1244	185 (5.2)	169 (17.1)	≤0.001	400 (10.9)	490 (26.2)	≤0.001
History of ischemic heart disease ^b^	1098	202 (5.6)	200 (20.3)	≤0.001	247 (6.7)	449 (24.0)	≤0.001
Alcohol drinker^b^	210	179 (5.0)	31 (3.1)	0.01	0 (0.0)	0 (0.0)	–
Active smoking^b^	2735	2025 (56.6)	432 (43.8)	≤0.001	133 (3.6)	145 (7.7)	≤0.001
Supplement use^b^	1647	382 (10.7)	74 (7.5)	0.003	871 (23.7)	320 (17.1)	≤0.001
Obesity status ^b^				≤0.001			≤0.001
Underweight BMI<18.4)	576	396 (11.1)	28 (2.9)		128 (3.5)	24 (1.3)	
Normal weight (BMI = 18.5–24.9)	4119	1890 (53.0)	355 (36.2)		1371 (37.4)	503 (27.0)	
overweight (BMI = 25–29.9)	3613	1033 (29.0)	417 (42.5)		1413 (38.5)	750 (40.2)	
obese (BMI ≥ 30)	5474	247 (6.9)	181 (18.5)		757 (20.6)	589 (31.6)	
Family history of diabetes^b^	4498	1397 (39.0)	430 (43.6)	0.009	1754 (47.7)	917 (49.0)	0.37
Family history of hypertension^b^	6394	2008 (56.1)	651 (66.0)	≤0.001	2351 (64.0)	1384 (73.9)	≤0.001
Family history of ischemic heart disease^b^	5369	1699 (47.5)	456 (46.2)	0.49	2127 (57.9)	1087 (58.1)	0.89
DQI-I score^a^	54.77 ± 11.66	54.62 ± 11.40	58.33 ± 11.75	≤0.001	53.95 ± 11.67	54.79 ± 11.76	0.01
HEI-2015 score^a^	50.00 ± 11.41	48.77 ± 10.85	51.99 ± 11.54	≤0.001	49.72 ± 11.57	51.83 ± 11.72	≤0.001
DDS score^a^	4.79 ± 1.96	5.20 ± 1.90	5.26 ± 1.95	0.40	4.23 ± 1.91	4.51 ± 1.88	≤0.001
MDS^a^	4.50 ± 1.52	4.29 ± 1.50	4.44 ± 1.50	0.008	4.58 ± 1.50	4.74 ± 1.56	≤0.001

Quantitative variables are reported as mean ± standard deviation and for qualitative as frequency (percentage)

^a^Independent samples t test, ^b^ χ2 test

*DDS* dietary diversity score, *MDS* Mediterranean dietary score, *DQI-I* diet quality index-international, *HEI-2015* healthy eating index-2015, *BMI* Body mass index, *SBP* Systolic blood pressure, *DBP* diastolic blood pressure, *WHR* waist to hip ratio

The characteristics of study participants among different quartiles of dietary indices stratified by hypertension status are reported in supplemental Tables 1, 2, 3 and 4. Both subjects with and without hypertension in the top quartile of MDS, compared with those in the first quartile, were more likely to have higher height, physical activity, be male, and be active smoker, while, they were less educated, and had a fewer prevalence for diabetes and dietary supplements use (*P* < 0.05). In contrast to hypertensive subjects, normotensive individuals in the top quartile of MDS had a lower age, family history of diabetes and hypertension, and a healthier profile for obesity measures (weight, WC, BMI, and HC) (*P* < 0.05) (supplemental Table 1). Both normotensive and hypertensive individuals with greater adherence to HEI-2015 were younger, had lower WC and obesity rates, diabetes, and ischemic heart disease, in comparison to those from lower levels (P < 0.05). In hypertensive patients, there were more subjects with a family history of hypertension among those with the highest scores of HEI-2015 than those with the lowest scores (*P* = 0.04). In contradiction of hypertensive participants, normotensive people in the highest HEI-2015 quartile had lower weight, HC, and BMI, while were more likely to have a family history of diabetes as well as to be physically active, male, and smoker, than those in lower quartiles (*P* < 0.05) (supplemental Table 2). For DDS, it was identified that, irrespective to hypertension status, with increase in adherence to DDS, the levels of education, height, obesity measures (weight, WC, BMI, and HC), physical activity, and the frequency of males, smoker and married participants increased, but, age and DBP decreased (*P* < 0.05). For subjects without hypertension, higher score for DDS was significantly related to a lower frequency of alcohol drinking and supplement use (P < 0.05). In hypertensive patients, there were less subjects with a family history of ischemic heart disease among those with the highest scores of DDS than those with the lowest scores (*P* ≤ 0/001) (supplemental Table 3). Moreover, regardless of hypertension status, increase in DQI-I was significantly related to a lower education level and a reduction in diabetes prevalence, obesity rate and obesity-related parameters (weight, WC, BMI, and HC). It was also observed that in normotensive people, higher score for DQI-I was significantly related to a lower systolic and diastolic BP and a decreased likelihood to be married, but the rate of smoking increased (*P* < 0.05). Hypertensive patients with higher adherence to the DQI-I had significantly lower physical activity level, compared with those with a low adherence (supplemental Table 4).

Multivariable-adjusted odds ratio and 95% CI for hypertension across quartiles of dietary indices for the whole population, males, and females are presented in Table 2. In the analysis of the whole population, after controlling for potential covariates including daily energy intake, age, gender, physical activity, smoking, family history of hypertension, BMI, and the level of education, a significant negative association was identified between MDS (OR: 0.86, 95%CI = 0.75–0.99; P-trend = 0.03) and HEI-2015 (OR: 0.79, 95%CI = 0.68–0.90; P-trend ≤0.001) with the risk of hypertension in the highest quartile compared with the lowest quartile. In addition, in the stratified analysis by gender, HEI-2015 remained as a significant preventive dietary approach against hypertension in both males (OR: 0.80, 95%CI = 0.64–0.99; P-trend = 0.05) and females (OR: 0.78, 95%CI = 0.66–0.94; P-trend = 0.04). The relation of MDS to hypertension was disappeared in the fully adjusted model for males and females. However, it remained significant in the model adjusted for daily energy intake and age (males: OR: 0.72, 95%CI = 0.58–0.89; P-trend = 0.008, females: OR: 0.80, 95%CI = 0.68–0.95; P-trend = 0.01). No significant relationship was detected between DQI-I and DDS with the odds of hypertension in the overall analysis and the stratified analysis by gender.

**Table 2:** Logistic regression analysis for the relationship between diet quality indices and the risk of hypertension in the whole population (*N* = 10,112), males (N = 4565), and females (N = 5547)

whole population			Model 1		Model 2		Model 3	
			OR (95% CI)	*P*-value	OR (95% CI)	*P*-value	OR (95% CI)	*P*-value
	MDS	Quartile 1	1		1		1	
		Quartile 2	0.94 (0.83–1.06)	0.35	0.97 (0.85–1.11)	0.73	1.03 (0.90–1.18)	0.63
		Quartile 3	0.80 (0.71–0.91)	0.001	0.81 (0.71–0.93)	0.003	0.88 (0.77–1.01)	0.08
		Quartile 4	0.79 (0.70–0.89)	≤0.001	0.76 (0.67–0.87)	≤0.001	0.86 (0.75–0.99)	0.03
		P -trend		≤0.001		≤0.001		0.03
	HEI-2015	Quartile 1	1		1		1	
		Quartile 2	0.53 (0.47–0.60)	≤0.001	0.83 (0.73–0.95)	0.007	0.91 (0.79–1.04)	0.17
		Quartile 3	0.62 (0.55–0.69)	≤0.001	0.72 (0.63–0.81)	≤0.001	0.80 (0.70–0.91)	0.001
		Quartile 4	0.75 (0.66–0.85)	≤0.001	0.66 (0.58–0.75)	≤0.001	0.79 (0.68–0.90)	0.001
		P -trend		≤0.001		≤0.001		≤0.001
	DDS	Quartile 1	1		1		1	
		Quartile 2	0.91 (0.81–1.03)	0.15	1.01 (0.89–1.15)	0.78	0.97 (0.84–1.11)	0.65
		Quartile 3	0.95 (0.84–1.08)	0.49	1.08 (0.94–1.23)	0.25	0.95 (0.82–1.09)	0.48
		Quartile 4	0.86 (0.75–0.99)	0.04	1.07 (0.92–1.24)	0.37	0.93 (0.79–1.09)	0.39
		P -trend		0.19		0.65		0.84
	DQI-I	Quartile 1	1		1		1	
		Quartile 2	0.83 (0.62–1.13)	0.25	0.87 (0.64–1.18)	0.39	0.86 (0.63–1.18)	0.37
		Quartile 3	0.80 (0.57–1.11)	0.19	0.83 (0.60–1.17)	0.29	0.87 (0.62–1.24)	0.46
		Quartile 4	0.76 (0.53–1.09)	0.14	0.83 (0.57–1.19)	0.31	0.90 (0.61–1.31)	0.58
		P -trend		0.41		0.67		0.81
Males	MDS	Quartile 1	1		1		1	
		Quartile 2	0.92 (0.75–1.13)	0.47	0.94 (0.76–1.17)	0.61	0.99 (0.79–1.24)	0.97
		Quartile 3	0.82 (0.67–1.01)	0.06	0.80 (0.64–0.99)	0.04	0.88 (0.71–1.11)	0.30
		Quartile 4	0.77 (0.63–0.93)	0.01	0.72 (0.58–0.89)	0.002	0.88 (0.70–1.09)	0.25
		P -trend		0.04		0.008		0.50
	HEI-2015	Quartile 1	1		1		1	
		Quartile 2	0.71 (0.58–0.87)	0.001	0.78 (0.63–0.96)	0.01	0.89 (0.72–1.11)	0.33
		Quartile 3	0.52 (0.42–0.63)	≤0.001	0.60 (0.49–0.74)	0.001	0.75 (0.60–0.93)	0.009
		Quartile 4	0.48 (0.39–0.59)	≤0.001	0.59 (0.48–0.73)	0.001	0.80 (0.64–0.99)	0.05
		P -trend		≤0.001		≤0.001		0.05
	DDS	Quartile 1	1		1		1	
		Quartile 2	1.02 (0.82–1.28)	0.82	1.07 (0.87–1.39)	0.39	0.99 (0.77–1.27)	0.96
		Quartile 3	1.19 (0.95–1.49)	0.11	1.26 (0.99–1.60)	0.06	1.05 (0.82–1.34)	0.69
		Quartile 4	1.08 (0.86–1.37)	0.47	1.25 (0.98–1.59)	0.06	1.00 (0.77–1.30)	0.97
		P -trend		0.35		0.16		0.95
	DQI-I	Quartile 1	1		1		1	
		Quartile 2	0.72 (0.42–1.21)	0.22	0.81 (0.47–1.40)	0.46	0.81 (0.45–1.46)	0.49
		Quartile 3	0.50 (0.27–0.92)	0.02	0.54 (0.29–1.01)	0.06	0.68 (0.34–1.35)	0.27
		Quartile 4	0.69 (0.34–1.39)	0.30	0.81 (0.39–1.68)	0.58	0.96 (0.42–2.20)	0.94
		P -trend		0.16		0.30		0.70
Females	MDS	Quartile 1	1		1		1	
		Quartile 2	0.98 (0.84–1.14)	0.81	0.99 (0.84–1.17)	0.98	1.05 (0.89–1.25)	0.51
		Quartile 3	0.82 (0.70–0.96)	0.01	0.83 (0.70–0.98)	0.03	0.89 (0.75–1.07)	0.22
		Quartile 4	0.86 (0.73–1.01)	0.06	0.80 (0.68–0.95)	0.01	0.87 (0.73–1.04)	0.14
		P -trend		0.04		0.01		0.13
	HEI-2015	Quartile 1	1		1		1	
		Quartile 2	0.79 (0.68–0.92)	≤0.001	0.87 (0.74–1.03)	0.11	0.92 (0.75–1.09)	0.35
		Quartile 3	0.72 (0.62–0.84)	≤0.001	0.80 (0.67–0.94)	0.008	0.84 (0.71–1.008)	0.06
		Quartile 4	0.60 (0.51–0.70)	≤0.001	0.71 (0.60–0.83)	0.001	0.78 (0.66–0.94)	0.008
		P -trend		0.004		0.001		0.04
	DDS	Quartile 1	1		1		1	
		Quartile 2	0.91 (0.79–1.06)	0.24	1.04 (0.89–1.22)	0.58	0.99 (0.84–1.18)	0.99
		Quartile 3	0.88 (0.75–1.03)	0.13	1.05 (0.88–1.24)	0.56	0.93 (0.78–1.12)	0.47
		Quartile 4	0.79 (0.66–0.96)	0.01	1.08 (0.88–1.32)	0.45	0.92 (0.74–1.14)	0.45
		P -trend		0.11		0.88		0.80
	DQI-I	Quartile 1	1		1		1	
		Quartile 2	0.89 (0.61–1.28)	0.54	0.91 (0.62–1.31)	0.61	0.89 (0.60–1.30)	0.56
		Quartile 3	0.94 (0.63–1.40)	0.78	0.97 (0.64–1.45)	0.88	1.00 (0.66–1.52)	0.97
		Quartile 4	0.77 (0.50–1.18)	0.24	0.82 (0.53–1.26)	0.37	0.86 (0.55–1.33)	0.50
		P -trend		0.69		0.81		0.84

*MDS* Mediterranean diet score, *HEI-2015* healthy eating index-2015, *DDS* diet diversity score, *DQI-I* diet quality index international

Model 1: Adjusted for daily energy intake

Model 2: Adjusted for daily energy intake, age, and gender

Model 3: Adjusted for daily energy intake, age, gender, physical activity, smoking, family history of hypertension, body mass index, and level of education

## Discussion

In the present study, we assessed the relation of four a priori defined diet quality indices, including MDS, DQI-I, HEI- 2015, and DDS to the risk of hypertension in a large population of Iranian adults. The findings revealed that people in the highest quartile of MDS and HEI-2015, compared with individuals in the lowest quartile, had significantly lower odds for having hypertension, but, no such relationship was observed for DQI-I and DDS. Furthermore, higher adherence to MDS, DQI-I, and HEI-2015 was associated with a significant decrease in obesity-related parameters including weight, WC, BMI, and HC. In contrast, greater score for DDS was related to an increase in obesity rates.

In the studied population of the present study, which included middle-aged/elderly people in Iran (aged from 35 to 70), hypertension was more prevalent among females than men. In line with our study, in a study in India, hypertension was more prevalent among Elderly females than men [30]. It is worth noting that the mean BMI (26.84 ± 4.86 vs. 24.19 ± 4.42) and WHR (94.88 ± 6.19 vs. 91.54 ± 6.33) were significantly higher in women compared with men in our study. Higher BMI and WHR have been strongly related to increased BP [31]; this might, in part, justify higher prevalence of hypertension observed in females in this study.

It has been proposed that high-quality diets, such as the MDS and HEI, may prevent development of hypertension in healthy people and could further reduces blood pressure in hypertensive people who use antihypertensive drugs [9, 32]. A recent meta-analysis found that intervention with the Mediterranean diet for 1 year decreased both SBP and DBP [33]. Supporting our results, the study by Shim et al. [9] reported that a high adherence to the Korean version of HEI is favorably related to BP control in men with physician-diagnosed hypertension. Moreover, the cross sectional study of Saraf-Bank et al. [34] revealed that a greater adherence to HEI-2010 is negatively liked to high BP among Iranian adult women. Nevertheless, the study by Daneshzad et al. [35] found no association between HEI and mean systolic and diastolic BP. Both MDS and HEI-2015 have many similarities and emphasize on high intake of fish, fruits, vegetables, monounsaturated fat, legumes, whole grains, and low consumption of saturated fatty acids. They also differ in some components such that the MDS scores dairies negatively but HEI-2015 scored diaries positively [26, 27]. Furthermore, HEI-2015 recommends lowers intakes for added sugars, refined grains, and sodium [26], which its association with hypertension is established [36]. These diet are rich in fiber and have a low energy density and low glycemic load, all of which are p[protective against hypertension [37]. The favorable impacts of vegetables/fruits on hypertension has been also demonstrated [38]. This relationship could also be attributed to potential healthy constituents such as phytochemicals, fibers, vitamins, magnesium, and potassium and antioxidants in vegetables and fruits [39], which have been independently related to a reduction in BP [40–42]. The study by Shah et al. [43] proposed lower inflammation, lower endothelial oxidative stress, and greater endothelial functioning as potential novel underlying mechanisms through which components of MDS might affects vascular changes related to improved cardiovascular risk.

There are very limited evidence regarding the relation of DDS and DQI-I to BP. Some previous studies reported lower systolic or diastolic BP in higher categorizes of DDS and DQI-I [21, 44]. A cross-sectional study of 82 adults living in Saba Island in the eastern Caribbean Basin identified a significant link between a diet with poor diversity and the increased risk of hypertension [44]. In contrast, a cross-sectional study on 230 Iranian women with type 2 diabetes identified no significant difference in SBD and DBP across categorizes of DQI-I [35]. In this study we found that, compared with individuals in the lowest quartile, people in the top quartile of the DDS had significantly lower mean of DBP, and for DQI-I, had a lower mean of both systolic and diastolic BP; nevertheless, multivariable adjusted logistic regression analysis found no significant association between DDS, DQI-I and hypertension. It should be considered that the results of logistic regression analysis is a more reliable as large sample size of the study makes small differences in means as significant; hence this study concluded a null association between DDS, DQI-I and risk of hypertension. The heterogeneity in the results of this study campared with the previous studies might be due to differences in sample size, method used to assess dietary intake, study design, health status of participants, different level of adjustment for confounders, and method of data analysis.

In line with our study, some studies [4, 21], but not all [45], reported that higher DDS is related to higher prevalence of obesity. This association is expectable as consumption of a more varied diet is linked to higher nutritional adequacy, greater intake of macronutrients and also energy [46], proposing that intake of a large number of foods may result in a higher calorie intake, and thus, obesity [21]. It is notable that the MDS, DQI-I, and HEI-2015 were related to a healthier profile for obesity measures, which has also been observed in some previous investigations [47–49]. These negative relationships with obesity parameters were expected, as these dietary patterns are rich in fiber and have low energy density and low glycemic load, all of which have a protective effect against obesity [50, 51]. However, the association of these dietary patterns on health outcomes is derived from the interaction of all components and could not be attributed to a single food item. The prospective study by Funtikova et al. [52] on 2181 participants revealed that, during a 10-year follow-up, a higher DQI score at baseline related to a decrease in WC. Another study on Chinese diabetic patients showed that the HEI, but not DQI-I, score had a negative relationship with obesity [53]. In a cross-sectional study recruiting 1062 Mexican women, with age between 35 to 69 years, MED was linked with lower WC whereas other a priori dietary patterns (DQI –I and HEI) were not predictors of anthropometric measures [54]. Moreover, unlike our findings, adherence to HEI, MDS, and DQI-I could not predict BMI and WC in Tehran Lipid and Glucose Study after 6.7 years of follow-up [55]. The capability of dietary indices to predict obesity measures relies on how well these indices correlate with dietary energy intake as the chief cause of obesity. However, these indices do not assign negative scores to excessive energy intakes; this limitation makes it difficult to reach a consistent conclusion across studies. The discrepancies regarding the relation of diet quality indices to obesity-related parameters may also be derived from differences in genetic background, interaction of gene-environmental factors, and difference in adjustment for potential confounders and disease pattern in various studies.

The strengths of the present study are its large population representative sample derived from a recent nationwide study and consideration of the potential covariates in the analysis. Moreover, another positive point of this study is face to face interview for data collection of FFQ, which makes the data more reliable. Also, conducting the anthropometric measurements instead of self-reporting can be considered as a strength. However, there are several limitations in this study that should be considered when inferring the findings. First, since our study had a cross-sectional design, causal association could not be inferred; although, cross-sectional studies provide valuable data on diet–disease associations. Future longitudinal studies can provide stronger evidence in this regard. Second, FFQ is susceptible to recall bias, such that subjects might under- or overestimate their food intake resulting in misclassification of food intake. As a structured dietary assessment method, the FFQ is less precise than methods of daily intake such as 24-h recalls and food records. Although 24-h food recall is used mostly in calculating HEI-2015, there are several well-designed studies that have used FFQ for calculating HEI-2015 [56]. Because of severe attenuation, both FFQ and multiple 24-h recalls cannot be recommended as an instrument for evaluating relations between absolute intake of diet and disease. These instruments could be used for detecting diet-disease association when considering the relative dietary intake of participants. Finally, however it was attempted to control for known covariates, the probability of residual confounding could not be omitted in our results.

## Conclusions

In conclusion, this study proposes that greater adherence to MDS and HEI-2015 diets, but not DQI-I and DDS, reduces the risk of hypertension. In addition, higher adherence to MDS, DQI-I, and HEI-2015 as well as lower score for DDS was related to a healthier profile for obesity measures. Further investigations, in particular prospective cohort studies, are required to confirm these conclusions.

## Supplementary Information

**Additional file 1: Supplemental Table 1**. Distribution of baseline variables based on the quartiles of Mediterranean diet score in subjects with and without hypertension. **Supplemental Table 2**. Distribution of baseline variables based on the quartiles of Healthy eating index-2015 score in subjects with and without hypertension. **Supplemental Table 3**. Distribution of baseline variables based on the quartiles of Diet diversity score in subjects with and without hypertension. **Supplemental Table 4**. Distribution of baseline variables based on the quartiles of Diet quality index-international score in subjects with and without hypertension.

## Data Availability

The datasets used and/or analyzed during the current study are available from the corresponding author by email.

## Abbreviations

CVD: Cardiovascular disease
DDS: Dietary diversity score
MDS: Mediterranean dietary score
DQI-I: Diet quality index-international
HEI-2015: healthy eating index-2015
DBP: Diastolic blood pressure
SBP: Systolic blood pressure
DASH: Dietary approach to stop hypertension
BP: Blood pressure
PERSIAN: the Prospective Epidemiological Research Study in Iran
FFQ: Food Frequency Questionnaire
MUFA/SFA: Monounsaturated fatty acids to saturated fatty acids
IPAQ: International Physical Activity Questionnaire
BMI: Body mass index
WC: Waist circumference
HC: Hip circumference
ANOVA: One-way analysis of variance
OR: Odds ratio
CI: Confidence interval
