# Supplementary material for: Diet quality in relation to the risk of hypertension among Iranian adults: cross-sectional analysis of Fasa PERSIAN cohort study
Source: Nutr J. 2021 Jun 26;20:57. doi: 10.1186/s12937-021-00717-1 (PMC8236133; doi:10.1186/s12937-021-00717-1)
Supplement: Supplementary file 1 — Additional file 1: Supplemental Table 1. Distribution of baseline variables based on the quartiles of Mediterranean diet score in subjects with and without hypertension. Supplemental Table 2. Distribution of baseline variables based on the quartiles of Healthy eating index-2015 score in subjects with and without hypertension. Supplemental Table 3. Distribution of baseline variables based on the quartiles of Diet diversity score in subjects with and without hypertension. Supplemental Table 4. Distribution of baseline variables based on the quartiles of Diet quality index-international score in subjects with and without hypertension. [file 12937_2021_717_MOESM1_ESM.docx]

|  | Without hypertension (n=7,254) | | | | | With hypertension (n=2,858)  Supplemental Table 1. Distribution of baseline variables based on the quartiles of Mediterranean diet score in subjects with and without hypertension. | | | | |
| --- | --- | --- | --- | --- | --- | --- | --- | --- | --- | --- |
|  | Mediterranean diet score | | | | | Mediterranean diet score | | | | |
|  | Quartile 1 | Quartile 2 | Quartile 3 | Quartile 4 | P-value^***^ | Quartile 1 | Quartile 2 | Quartile 3 | Quartile 4 | P-value^***^ |
| Age (year) | 8.93±46.81 | 8.76±46.15 | 8.73±46.43 | 8.97±47.10 | 0.007 | 9.30±53.55 | 9.45±53.59 | 9.55±53.71 | 9.52±53.92 | 0.87 |
| Education (year) | 4.03±5.36 | 3.80±5.44 | 3.84±5.16 | 7.33±4.80 | ≤0.001 | 3.78±3.67 | 3.50±3.39 | 3.45±3.17 | 3.66±3.14 | 0.01 |
| Height (cm) | 9.65±161.63 | 10.65±162.30 | 9.15±163.01 | 9.67±162.86 | ≤0.001 | 8.42±158.81 | 8.58±160.05 | 10.47±159.96 | 8.53±160.00 | 0.01 |
| Wight (kg) | 13.15±66.94 | 13.19±66.16 | 13.33±65.85 | 12.45±64.69 | ≤0.001 | 13.81±69.95 | 12.96±70.06 | 14.01±70.34 | 13.49±69.43 | 0.67 |
| WC (cm) | 11.37±92.63 | 11.34±91.67 | 11.37±90.98 | 11.48±89.91 | 0.001 | 11.32±98.58 | 10.81±97.99 | 11.47±97.83 | 11.61±97.28 | 0.17 |
| HC (cm) | 8.86±99.82 | 8.52±98.91 | 8.61±98.50 | 8.28±97.74 | ≤0.001 | 9.31±102.19 | 8.69±101.94 | 9.18±102.02 | 9.26±100.96 | 0.06 |
| BMI(kg/m2) | 4.68±25.61 | 4.75±25.08 | 4.61±24.78 | 4.67±24.41 | ≤0.001 | 4.87±27.69 | 4.55±27.34 | 4.93±27.37 | 4.83±27.10 | 0.11 |
| Physical activity(MET) | 10.53±40.45 | 11.79±41.74 | 11.77±42.67 | 12.53±43.95 | 0.001 | 8.83±38.40 | 10.09±39.71 | 11.09±40.40 | 9.63±39.69 | ≤0.001 |
| DBP (mmHg) | 8.98±70.66 | 9.04±70.76 | 8.83±70.46 | 8.84±70.11 | 0.12 | 12.60±85.08 | 12.23±84.73 | 12.41±85.37 | 11.93±85.73 | 0.48 |
| SBP (mmHg) | 82.49±104.12 | 22.34±104.12 | 53.20±104.12 | 99.57±103.12 | 0.19 | 20.23±128.63 | 19.30±128.85 | 19.72±129.00 | 18.86±129.95 | 0.59 |
| Sex (Male) | 762 (42.2) | 803 (48.6) | 925 (51.0) | 1089 (54/3) | ≤0.001 | 243 (28.8) | 236 (33.8) | 242 (37.8) | 265 (39.2) | ≤0.001 |
| Marital status |  |  |  |  | 0.08 |  |  |  |  | 0.30 |
| Single | 99 (5.5) | 65 (3.9) | 79 (4.4) | 70 (5/3) |  | 13 (1.5) | 18 (2.6) | 14 (2.2) | 16 (2.4) |  |
| Married | 1599 (88.6) | 1493 (90.3) | 1633 (90.1) | 1813 (4/91) |  | 710 (84.1) | 597 (85.5) | 563 (88.0) | 586 (86.7) |  |
| Widow | 94 (5.2) | 77 (4.7) | 79 (4.4) | 80 (0/4) |  | 112 (13.3) | 76 (10.9) | 56 (8.8) | 68 (10.1) |  |
| Divorced | 13 (0.7) | 18 (1.1) | 21 (1.2) | 21 (1/1) |  | 9 (1.1) | 7 (1.0) | 7 (1.1) | 6 (0.9) |  |
| History of diabetes | 183 (10.1) | 141 (8.5) | 141 (7.8) | 120 (0/6) | ≤0.001 | 231 (27.4) | 172 (24.6) | 122 (19.1) | 134 (19.4) | ≤0.001 |
| History of ischemic heart disease | 118 (6.5) | 101 (6.1) | 99 (5.5) | 131 (6/6) | 0.45 | 213 (25.2) | 162 (23.2) | 138 (21.6) | 136 (20.1) | 0.10 |
| Alcohol drinker | 33 (1.8) | 45 (2.7) | 45 (2.5) | 56(2.8) | 0.20 | 7 (0.8) | 5 (0.7) | 11 (1.7) | 8 (1.2) | 0.27 |
| Active smoking | 447 (24.8) | 483 (29.2) | 553 (30.5) | 675 (34.0) | ≤0.001 | 141 (16.7) | 130 (18.6) | 145 (22.7) | 161 (23.8) | 0.002 |
| Supplement use | 325 (18.0) | 316 (19.1) | 310 (17.1) | 302 (15.2) | 0.01 | 136 (16.1) | 107 (15.3) | 71 (11.1) | 80 (11.8) | 0.01 |
| Obesity status |  |  |  |  | ≤0.001 |  |  |  |  | 0.40 |
| Underweight BMI˂18.4) | 91 (5.1) | 119 (7.2) | 145 (8.0) | 169 (8.6) |  | 13 (1.5) | 8 (1.1) | 14 (2.2) | 17 (2.5) |  |
| Normal weight  (BMI=18.5-24.9) | 727 (40.4) | 737 (44.6) | 820 (45.4) | 977 (49.4) |  | 233 (27.8) | 216 (31.0) | 192 (30.1) | 217 (32.2) |  |
| overweight (BMI=25-29.9) | 687 (38.1) | 554 (33.6) | 606 (33.5) | 599 (30.3) |  | 349 (41.6) | 288 (41.3) | 262 (41.1) | 268 (39.8) |  |
| obese (BMI≥30) | 296 (16.4) | 241 (14.6) | 236 (13.1) | 231 (11.7) |  | 244 (29.1) | 185 (26.5) | 169 (26.5) | 172 (25.5) |  |
| Family history of diabetes | 824 (45.7) | 725 (43.9) | 818 (45.1) | 784 (39.5) | ≤0.001 | 411 (48.7) | 349 (50.0) | 285 (44.5) | 302 (44.7) | 0.08 |
| Family history of hypertension | 1110 (61.5) | 1012 (61.2) | 1109 (61.2) | 1128 (56.9) | 0.008 | 631 (74.8) | 492 (70.5) | 446 (69.7) | 466 (68.9) | 0.06 |
| Family history of ischemic heart disease | 943 (52.2) | 883 (53.4) | 979 (54.0) | 1021 (51.5) | 0.39 | 476 (56.4) | 380 (54.4) | 337 (52.7) | 350 (51.8) | 0.28 |

***χ2for trend.

Quantitative variables are reported as mean ± standard deviation and for qualitative as frequency (percentage).

WC: Waist circumferences; HC: Hip circumferences; BMI: Body mass index; SBP: Systolic blood pressure; DBP: diastolic blood pressure.

Supplemental Table 2. Distribution of baseline variables based on the quartiles of Healthy eating index-2015 score in subjects with and without hypertension.

|  | Without hypertension (n=7,254) | | | | | With hypertension (n=2,858) | | | | |
| --- | --- | --- | --- | --- | --- | --- | --- | --- | --- | --- |
|  | Healthy eating index-2015 score | | | | | Healthy eating index-2015 score | | | | |
|  | Quartile 1 | Quartile 2 | Quartile 3 | Quartile 4 | P-value^***^ | Quartile 1 | Quartile 2 | Quartile 3 | Quartile 4 | P-value^***^ |
| Age (year) | 9.00±47.83 | 8.99±46.59 | 8.82±46.33 | 8.55±45.96 | ≤0.001 | 9.26±54.45 | 9.26±53.80 | 9.90±53.00 | 9.39±53.12 | 0.009 |
| Education (year) | 4.01±5.25 | 3.84±5.19 | 3.81±5.23 | 3.82±5.04 | 0.36 | 3.72±3.60 | 3.56±3.30 | 3.66±3.18 | 3.48±3.27 | 0.10 |
| Height (cm) | 9.63±162.19 | 10.66±162.04 | 9.81±163.07 | 9.08±162.45 | 0.008 | 9.93±159.51 | 8.60±159.81 | 8.47±159.45 | 8.46±159.88 | 0.76 |
| Wight (kg) | 13.23±67.96 | 13.06±65.70 | 12.93±65.88 | 12.70±64.19 | ≤0.001 | 13.20±70.59 | 13.51±69.98 | 14.10±68.70 | 13.62±70.13 | 0.06 |
| WC (cm) | 11.19±93.57 | 11.08±91.29 | 11.42±90.65 | 11.66±89.81 | 0.001 | 10.69±98.95 | 11.16±98.06 | 11.86±96.51 | 11.65±97.79 | 0.001 |
| HC (cm) | 8.67±100.15 | 8.37±98.61 | 8.54±98.45 | 8.62±97.81 | ≤0.001 | 10.69±98.95 | 9.00±101.73 | 9.87±101.20 | 9.44±101.86 | 0.21 |
| BMI(kg/m2) | 4.62±25.80 | 4.63±24.98 | 4.69±24.77 | 4.73±24.38 | ≤0.001 | 4.65±27.66 | 4.74±27.37 | 5.03±26.99 | 4.84±27.42 | 0.07 |
| Physical activity(MET) | 11.28±41.43 | 11.55±41.98 | 11.87±42.50 | 12.20±42.96 | 0.001 | 9.15±38.98 | 10.75±39.89 | 9.86±39.47 | 10.00±39.75 | 0.26 |
| DBP (mmHg) | 8.61±71.32 | 8.93±70.44 | 9.02±70.19 | 9.03±70.08 | ≤0.001 | 12.72±84.79 | 11.92±85.38 | 12.39±85.42 | 12.05±85.45 | 0.65 |
| SBP (mmHg) | 12.17±105.78 | 12.16±104.28 | 12.57±104.19 | 12.57±103.43 | ≤0.001 | 19.86±129.19 | 19.86±128.78 | 19.52±128.61 | 18.91±129.65 | 0.77 |
| Sex (Male) | 783(45.7) | 785(48.1) | 1025(52.6) | 986(50.3) | ≤0.001 | 323(34.8) | 234(34.8) | 206(34.2) | 223(34.0) | ≤0.98 |
| Marital status |  |  |  |  | 0.004 |  |  |  |  | 0.06 |
| Single | 65(3.8) | 86(5.3) | 84(4.3) | 78(4.0) |  | 15(1.6) | 14(2.1) | 20(3.3) | 12(1.8) |  |
| Married | 1534(89.5) | 1469(90.1) | 1768(90.7) | 1767(90.2) |  | 780(84.1) | 588(87.5) | 526(87.2) | 562(85.7) |  |
| Widow | 105(6.1) | 59(3.6) | 75(3.8) | 91(4.6) |  | 121(13.1) | 64(9.5) | 54(9.0) | 73(11.1) |  |
| Divorced | 10(0.6) | 17(1.0) | 22(1.1) | 24(1.2) |  | 11(1.2) | 6(0.9) | 3(0.5) | 9(1.4) |  |
| History of diabetes | 224(13.1) | 134(8.2) | 124(6.4) | 103(5.3) | ≤0.001 | 264(28.5) | 151(22.5) | 106(17.6) | 138(21.0) | ≤0.001 |
| History of ischemic heart disease | 135(7.9) | 109(6.7) | 80(4.1) | 125(6.4) | ≤0.001 | 234(25.2) | 161(24.0) | 123(20.4 | 131(20.0) | 0.03 |
| Alcohol drinker | 35(2.0) | 39(2.4) | 56(2.9) | 49(2.5) | 0.44 | 9(1.0) | 6(0.9) | 12(2.0) | 4(0.6) | 0.09 |
| Active smoking | 411(24.0) | 461(28.3) | 639(32.8) | 647(33.0) | ≤0.001 | 175(18.9) | 136(20.2) | 135(22.4) | 131(20.0) | 0.42 |
| Supplement use | 131(7.6) | 113(6.9) | 105(5.4) | 108(5.5) | 0.66 | 250(27.0) | 143(21.3) | 91(15.1) | 117(17.8) | 0.34 |
| Obesity status |  |  |  |  | ≤0.001 |  |  |  |  | 0.03 |
| Underweight BMI˂18.4) | 82(4.8) | 109(6.7) | 142(7.3) | 191(9.8) |  | 15(1.6) | 8(1.2) | 19(3.2) | 10(1.5) |  |
| Normal weight  (BMI=18.5-24.9) | 666(39.0) | 729(44.8) | 920(47.3) | 946(48.4) |  | 249(27.1) | 208(31.0) | 204(33.9) | 197(30.1) |  |
| overweight (BMI=25-29.9) | 678(39.7) | 568(34.9) | 617(31.7) | 583(29.8) |  | 391(42.5) | 278(41.5) | 229(38.0) | 269(41.1) |  |
| obese (BMI≥30) | 281(16.5) | 222(13.6) | 266(13.7) | 235(12.0) |  | 265(28.8) | 176(26.3) | 150(24.9) | 179(27.3) |  |
| Family history of diabetes | 814(47.5) | 714(43.8) | 810(41.6) | 813(41.5) | 0.001 | 462(49.8) | 311(46.3) | 277(45.9) | 297(45.3) | 0.24 |
| Family history of hypertension | 1035(60.4) | 995(61.0) | 1182(60.6) | 1147(58.5) | 0.40 | 679(73.2) | 489(72.8) | 427(70.8) | 440(67.1) | 0.04 |
| Family history of ischemic heart disease | 932(54.4) | 855(52.4) | 1029(52.8) | 1010(51.5) | 0.38 | 514(55.4) | 365(54.3) | 319(52.9) | 345(52.6) | 0.65 |

***χ2for trend.

Quantitative variables are reported as mean ± standard deviation and for qualitative as frequency (percentage).

WC: Waist circumferences; HC: Hip circumferences; BMI: Body mass index; SBP: Systolic blood pressure; DBP: diastolic blood pressure.

|  | Without hypertension (n=7,254) | | | | | With hypertension (n=2,858)  Supplemental Table 3. Distribution of baseline variables based on the quartiles of Diet diversity score in subjects with and without hypertension. | | | | |
| --- | --- | --- | --- | --- | --- | --- | --- | --- | --- | --- |
|  | Diet diversity score | | | | | Diet diversity score | | | | |
|  | Quartile 1 | Quartile 2 | Quartile 3 | Quartile 4 | P-value^***^ | Quartile 1 | Quartile 2 | Quartile 3 | Quartile 4 | P-value^***^ |
| Age (year) | 9.02±48.07 | 8.77±46.91 | 9.02±46.45 | 8.42±45.29 | ≤0.001 | 9.16±55.86 | 9.00±53.69 | 9.68±53.21 | 9.43±51.31 | ≤0.001 |
| Education (year) | 3.60±4.22 | 3.82±4.94 | 3.80±5.29 | 3.98±6.14 | ≤0.001 | 2.90±2.26 | 3.44±3.25 | 3.65±3.52 | 4.10±4.80 | ≤0.001 |
| Height (cm) | 8.75±160.03 | 10.22±161.82 | 8.91±162.72 | 10.39±165.01 | ≤0.001 | 7.72±157.21 | 9.83±158.94 | 8.52±160.40 | 9.01±162.91 | ≤0.001 |
| Wight (kg) | 12.40±62.60 | 12.64±65.04 | 12.91±66.76 | 13.35±68.77 | ≤0.001 | 13.08±66.00 | 12.78±68.98 | 13.63±71.80 | 13.48±74.20 | ≤0.001 |
| WC (cm) | 11.78±90.43 | 11.31±91.07 | 11.38±91.80 | 11.25±91.66 | 0.001 | 11.74±96.74 | 10.95±97.95 | 11.48±98.53 | 10.79±98.93 | 0.001 |
| HC (cm) | 8.80±97.60 | 8.28±98.33 | 8.61±99.17 | 8.56±99.66 | ≤0.001 | 9.52±100.19 | 8.85±101.50 | 9.23±102.73 | 8.43±103.22 | ≤0.001 |
| BMI(kg/m2) | 4.73±24.49 | 4.67±24.80 | 4.63±25.24 | 4.73±25.24 | ≤0.001 | 4.83±26.67 | 4.68±27.22 | 4.89±27.91 | 4.67±27.96 | ≤0.001 |
| Physical activity(MET) | 10.89±41.65 | 11.25±41.88 | 11.71±42.25 | 12.94±43.15 | 0.001 | 8.55±38.30 | 9.64±39.55 | 9.58±39.58 | 11.61±40.84 | ≤0.001 |
| DBP (mmHg) | 8.76±71.00 | 8.83±70.65 | 8.96±70.22 | 9.12±70.02 | 0.004 | 11.78±86.77 | 11.62±85.20 | 12.05±85.55 | 13.32±83.76 | ≤0.001 |
| SBP (mmHg) | 13.02±104.34 | 12.58±104.14 | 12.20±104.25 | 11.87±104.74 | 0.47 | 20.88±128.77 | 19.74±129.65 | 18.23±128.65 | 19.08±129.31 | 0.74 |
| Sex (Male) | 643 (37.4) | 852 (47.0) | 888 (49.1) | 1196 (62.5) | ≤0.001 | 173 (21.3) | 228 (31.4) | 270 (37.9) | 315 (51.9) | ≤0.001 |
| Marital status |  |  |  |  | ≤0.001 |  |  |  |  | ≤0.001 |
| Single | 93 (5.4) | 84 (4.6) | 68 (3.8) | 68 (3.6) |  | 20 (2.5) | 19 (2.6) | 12 (1.7) | 10 (1.6) |  |
| Married | 1449 (84.3) | 1630 (90.0) | 1654 (91.4) | 1805 (94.3) |  | 649 (79.7) | 619 (85.4) | 626 (87.9) | 562 (92.6) |  |
| Widow | 147 (8.6) | 79 (4.4) | 71 (3.9) | 33 (1.7) |  | 130 (16.0) | 86 (11.9) | 65 (9.1) | 31 (5.1) |  |
| Divorced | 29 (1.7) | 19 (1.0) | 16 (0.9) | 9 (0.5) |  | 15 (1.8) | 1 (0.1) | 9 (1.3) | 4 (0.7) |  |
| History of diabetes | 156 (9.1) | 166 (9.2) | 130 (7.2) | 133 (6.9) | 0.01 | 191 (23.5) | 183 (25.2) | 178 (25.0) | 107 (17.6) | 0.03 |
| History of ischemic heart disease | 129 (7.5) | 101 (5.6) | 106 (5.9) | 113 (5.9) | 0.07 | 237 (29.1) | 153 (21.1) | 148 (20.8) | 111 (18.3) | ≤0.001 |
| Alcohol drinker | 24 (1.4) | 30 (1.7) | 45 (2.5) | 80 (4.2) | ≤0.001 | 5 (0.6) | 8 (1.1) | 10 (1.4) | 8 (1.3) | 0.44 |
| Active smoking | 445 (25.9) | 505 (27.9) | 534 (29.5) | 674 (35.2) | ≤0.001 | 129 (15.8) | 144 (19.9) | 145 (20.4) | 159 (26.2) | ≤0.001 |
| Supplement use | 259 (15.1) | 333 (18.4) | 309 (17.1) | 352 (18.4) | 0.02 | 115 (14.1) | 90 (12.4) | 103 (14.5) | 86 (14.2) | 0.66 |
| Obesity status |  |  |  |  | ≤0.001 |  |  |  |  | ≤0.001 |
| Underweight BMI˂18.4) | 171 (10.0) | 130 (7.2) | 113 (6.3) | 110 (5.8) |  | 26 (3.2) | 14 (1.9) | 6 (0.8) | 6 (1.0) |  |
| Normal weight  (BMI=18.5-24.9) | 802 (46.7) | 849 (47.0) | 779 (43.3) | 831 (43.4) |  | 288 (35.4) | 218 (30.2) | 192 (27.0) | 160 (26.5) |  |
| overweight (BMI=25-29.9) | 534 (31.1) | 581 (32.2) | 648 (36.0) | 683 (35.7) |  | 318 (39.1) | 303 (42.0) | 301 (42.4) | 245 (40.6) |  |
| obese (BMI≥30) | 210 (12.2) | 245 (13.6) | 260 (14.4) | 289 (15.1) |  | 181 (22.1) | 186 (25.8) | 211 (29.7) | 192 (31.8) |  |
| Family history of diabetes | 731 (42.5) | 777 (42.9) | 811 (44.8) | 832 (43.4) | 0.53 | 373 (45.8) | 360 (49.7) | 340 (47.8) | 274 (45.1) | 0.32 |
| Family history of hypertension | 1024 (59.6) | 1085 (59.9) | 1095 (60.5) | 1155 (60.3) | 0.94 | 579 (71.1) | 515 (71.0) | 523 (73.5) | 418 (68.9) | 0.33 |
| Family history of ischemic heart disease | 913 (53.1) | 972 (53.6) | 955 (52.8) | 986 (51.5) | 0.59 | 437 (53.7) | 413 (57.0) | 386 (54.2) | 307 (50.6) | 0.14 |

***χ2for trend.

Quantitative variables are reported as mean ± standard deviation and for qualitative as frequency (percentage).

WC: Waist circumferences; HC: Hip circumferences; BMI: Body mass index; SBP: Systolic blood pressure; DBP: diastolic blood pressure.

|  | Without hypertension (n=7,254) | | | | | With hypertension (n=2,858)  Supplemental Table 4. Distribution of baseline variables based on the quartiles of Diet quality index-international score in subjects with and without hypertension. | | | | |
| --- | --- | --- | --- | --- | --- | --- | --- | --- | --- | --- |
|  | Diet quality index-international | | | | | Diet quality index-international | | | | |
|  | Quartile 1 | Quartile 2 | Quartile 1 | Quartile 2 | Quartile 1 | Quartile 2 | Quartile 1 | Quartile 2 | Quartile 1 | Quartile 2 |
| Age (year) | 8.60±47.12 | 8.79±46.39 | 9.02±46.53 | 8.98±46.60 | 0.08 | 9.16±53.27 | 9.46±53.50 | 9.44±53.81 | 9.75±54.30 | 0.19 |
| Education (year) | 3.83±5.32 | 3.89±5.27 | 3.86±5.13 | 3.87±4.99 | 0.04 | 3.92±3.99 | 3.37±3.39 | 3.67±3.23 | 3.31±2.69 | ≤0.001 |
| Height (cm) | 8.88±163.13 | 10.83±162.91 | 9.90±162.26 | 9.19±161.62 | ≤0.001 | 10.30±160.94 | 8.28±160.07 | 8.60±159.21 | 8.15±157.97 | ≤0.001 |
| Wight (kg) | 13.06±69.11 | 13.12±67.09 | 12.64±64.89 | 12.51±62.80 | ≤0.001 | 13.31±72.21 | 13.65±71.09 | 13.57±69.18 | 13.08±66.50 | ≤0.001 |
| WC (cm) | 10.87±93.71 | 11.36±91.91 | 11.28±90.51 | 11.68±89.19 | ≤0.001 | 10.70±98.96 | 11.44±98.73 | 11.22±97.66 | 11.75±96.08 | ≤0.001 |
| HC (cm) | 8.44±100.40 | 8.68±99.39 | 8.25±98.01 | 8.64±97.26 | ≤0.001 | 8.32±102.44 | 9.28±102.45 | 9.22±101.57 | 9.66±100.44 | ≤0.001 |
| BMI(kg/m2) | 4.50±25.97 | 4.73±25.21 | 4.66±24.67 | 4.70±24.10 | ≤0.001 | 4.58±27.79 | 4.92±27.73 | 4.77±27.26 | 4.87±26.64 | ≤0.001 |
| Physical activity(MET) | 11.75±42.26 | 11.80±42.20 | 11.91±42.52 | 11.61±42.05 | 0.67 | 10.73±40.15 | 10.15±39.71 | 4.77±27.26 | 8.49±38.80 | 0.03 |
| DBP (mmHg) | 8.53±71.42 | 8.87±70.77 | 8.94±70.23 | 9.19±69.62 | ≤0.001 | 12.40±85.95 | 12.33±85.05 | 11.80±84.82 | 12.64±84.89 | 0.25 |
| SBP (mmHg) | 11.76±105.60 | 12.20±104.72 | 12.60±104.33 | 12.84±103.05 | ≤0.001 | 19.13±129.62 | 19.88±128.72 | 19.53±129.08 | 19.79±128.83 | 0.80 |
| Sex (Male) | 815 (50.6) | 994 (50.7) | 883 (49.2) | 887 (47.0) | 0.08 | 350 (43.6) | 271 (35.1) | 198 (30.9) | 167 (26.0) | ≤0.001 |
| Marital status |  |  |  |  | 0.007 |  |  |  |  | 0.09 |
| Single | 61 (3.8) | 75 (3.8) | 88 (4.9) | 89 (4.7) |  | 14 (1.7) | 14 (1.8) | 17 (2.7) | 16 (2.5) |  |
| Married | 1473 (91.5) | 1789 (91.2) | 1609 (89.6) | 1667 (88.3) |  | 716 (89.3) | 665 (86.0) | 530 (82.7) | 545 (84.9) |  |
| Widow | 65 (4.0) | 80 (4.1) | 86 (4.8) | 99 (5.2) |  | 65 (8.1) | 85 (11.0) | 87 (13.6) | 75 (11.7) |  |
| Divorced | 11 (0.7) | 18 (0.9) | 12 (0.7) | 32 (1.7) |  | 7 (0.9) | 9 (1.2) | 7 (1.1) | 6 (0.9) |  |
| History of diabetes | 188 (11.7) | 171 (8.7) | 125 (7.0) | 101 (5.4) | ≤0.001 | 213 (26.6) | 184 (23.8) | 144 (22.5) | 118 (18.4) | 0.003 |
| History of ischemic heart disease | 113 (7.0) | 105 (5.4) | 113 (6.3) | 118 (6.3) | 0.22 | 183 (22.8) | 192 (24.8) | 142 (22.2) | 132 (20.6) | 0.28 |
| Alcohol drinker | 36 (2.2) | 54 (2.8) | 45 (2.5) | 44 (2.3) | 0.75 | 9 (1.2) | 9 (1.2) | 8 (1.2) | 5 (0.8) | 0.85 |
| Active smoking | 423 (26.3) | 564 (28.7) | 541 (30.1) | 630 (33.4) | ≤0.001 | 166 (20.7) | 170 (22.0) | 115 (17.9) | 126 (19.6) | 0.28 |
| Supplement use | 101 (6.3) | 134 (6.8) | 105 (5.8) | 117 (6.2) | 0.62 | 184 (22.9) | 171 (22.1) | 129 (20.1) | 117 (18.2) | 0.39 |
| Obesity status |  |  |  |  | ≤0.001 |  |  |  |  | ≤0.001 |
| Underweight BMI˂18.4) | 60 (3.7) | 119 (6.1) | 153 (8.5) | 192 (10.2) |  | 9 (1.1) | 11 (1.4) | 14 (2.2) | 18 (2.8) |  |
| Normal weight  (BMI=18.5-24.9) | 622 (38.8) | 858 (43.8) | 818 (45.7) | 963 (51.1) |  | 201 (25.2) | 225 (29.3) | 197 (30.8) | 235 (36.7) |  |
| overweight (BMI=25-29.9) | 657 (41.0) | 685 (35.0) | 589 (32.9) | 515 (27.3) |  | 359 (44.9) | 310 (40.4) | 256 (40.1) | 242 (37.8) |  |
| obese (BMI≥30) | 264 (16.5) | 296 (15.1) | 230 (12.8) | 214 (11.4) |  | 230 (28.8) | 222 (28.9) | 172 (26.9) | 146 (22.8) |  |
| Family history of diabetes | 746 (46.3) | 858 (43.7) | 745 (41.5) | 802 (42.5) | 0.02 | 393 (49.0) | 392 (50.7) | 270 (42.1) | 292 (45.5) | 0.007 |
| Family history of hypertension | 985 (61.2) | 1195 (60.9) | 1069 (59.6) | 1110 (58.8) | 0.42 | 579 (72.2) | 555 (71.8) | 456 (71.1) | 445 (69.3) | 0.65 |
| Family history of ischemic heart disease | 876 (54.4) | 1022 (52.1) | 947 (52.8) | 981 (52.0) | 0.46 | 443 (55.2) | 424 (54.9) | 342 (53.4) | 334 (52.0) | 0.60 |

Quantitative variables are reported as mean ± standard deviation and for qualitative as frequency (percentage).

WC: Waist circumferences; HC: Hip circumferences; BMI: Body mass index; SBP: Systolic blood pressure; DBP: diastolic blood pressure.
